# Supplementary material for: Astrocyte allocation during brain development is controlled by Tcf4-mediated fate restriction
Source: EMBO J. 2024 Sep 19;43(21):5114–40. doi: 10.1038/s44318-024-00218-x (PMC11535398; doi:10.1038/s44318-024-00218-x)
Supplement: Supplementary file 1 — Appendix [file 44318_2024_218_MOESM1_ESM.pdf]

APPENDIX

Astrocyte allocation during brain development is controlled by Tcf4-mediated fate restriction

Yandong Zhang[1#], Dan Li[1#], Yuqun Cai[1#], Rui Zou[1], Yilan Zhang[1], Xin Deng[1], Yafei Wang[1], Tianxiang Tang[1], Yuanyuan Ma[1], Feizhen Wu[2, 3], Yunli Xie[1,*]

TABLE OF CONTENTS

Appendix Figure S1. The expression of Tcf4 in MGE at different developmental stages. Page 2

Appendix Figure S2. The oligodendrocyte networks in the neocortex of Tcf4 cKO brains remain unchanged compared to those in WT brains. Page 3

Appendix Figure S3. The density of Sox9-positive and Olig2-positive cells in the ventral brain of Tcf4 cKO mice are unchanged compared to those in WT mice. Page 4

Appendix Figure S4. Quality control of single-cell RNA sequencing data from the Nkx2.1 lineage in the dorsal neocortex. Page 5

Appendix Figure S5. Depletion of Tcf4 in Nkx2.1-expressing RGCs enhances the accessibility of chromatin regions associated with genes related to astrogenesis. Page 7

Appendix Figure S6. Deletion of Tcf4 in Nkx2.1-expressing RGCs alters epigenetic states of enhancers. Page 8

Appendix Figure S7. Design of Cre-dependent Tcf4-tag mice. Page 10

Appendix Figure S1

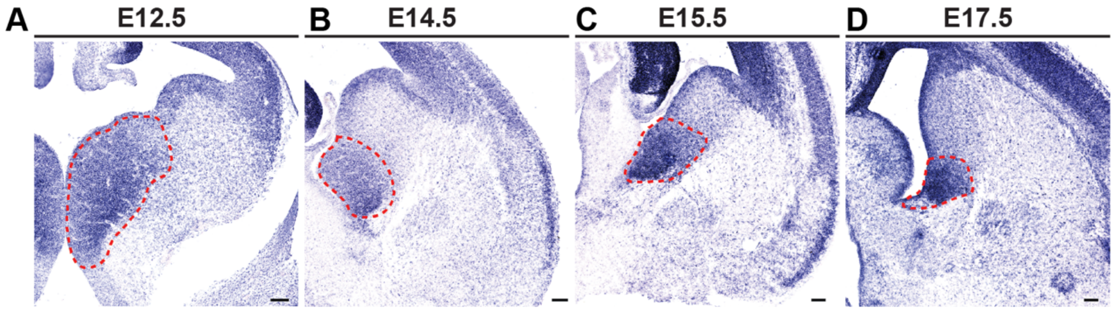

Appendix Figure S1. The expression of Tcf4 in MGE at different developmental stages. Expression of *Tcf4* in the MGE from early to later developmental stages (A-D) was examined by *in situ* hybridization in WT brains. The MGE region is outlined with a red dashed line; scale bar: 100 µm.

Appendix Figure S2

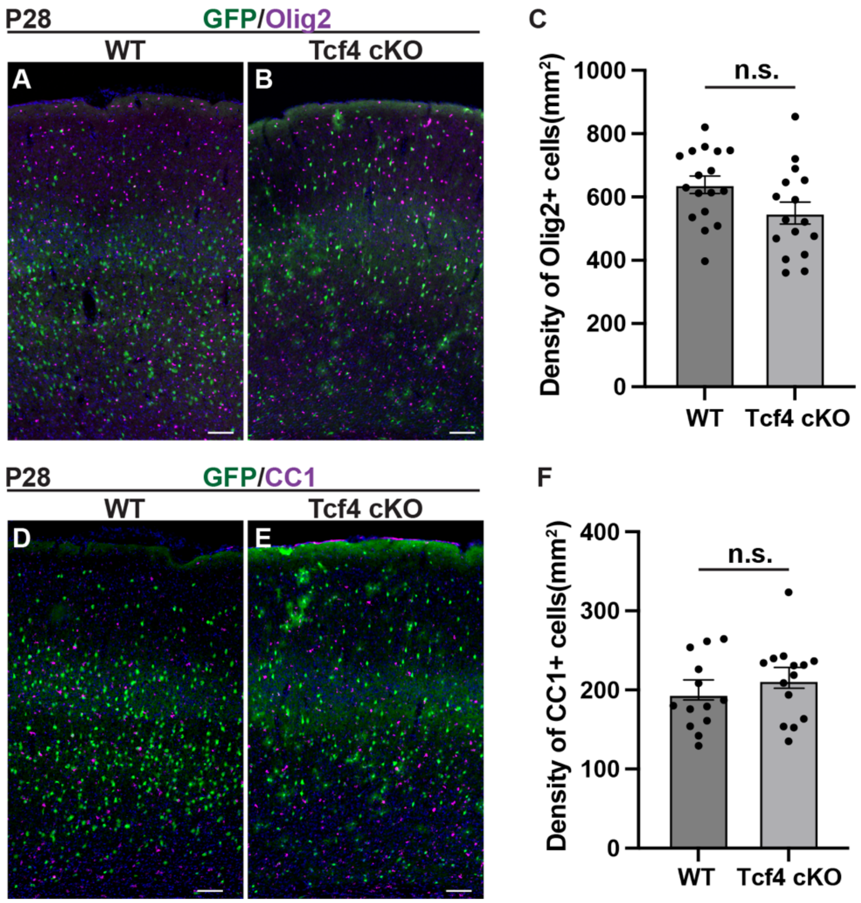

Appendix Figure S2. The oligodendrocyte networks in the neocortex of Tcf4 cKO brains remain unchanged compared to those in WT brains. (A-B) Representative images of brain sections stained for Olig2 and GFP in WT brain (A) and Tcf4 cKO (B) at P28. Scale bar: 100 μm. (C) Quantification of the density of the Olig2+ cells in the neocortex at P28 (n = 3 mice analyzed for each genotype). (D-E) Representative images of brain sections stained for CC1 and GFP in WT brain (D) and Tcf4 cKO (E) at P28. Scale bar: 100 µm. (F)Quantification of the density of the CC1+ cells in the neocortex at P28 (n = 3 mice analyzed for each genotype). Comparison between the two groups was conducted using a *t*-test within a linear mixed model. Error bars represent mean ± SEM. n.s., not significant.

Appendix Figure S3

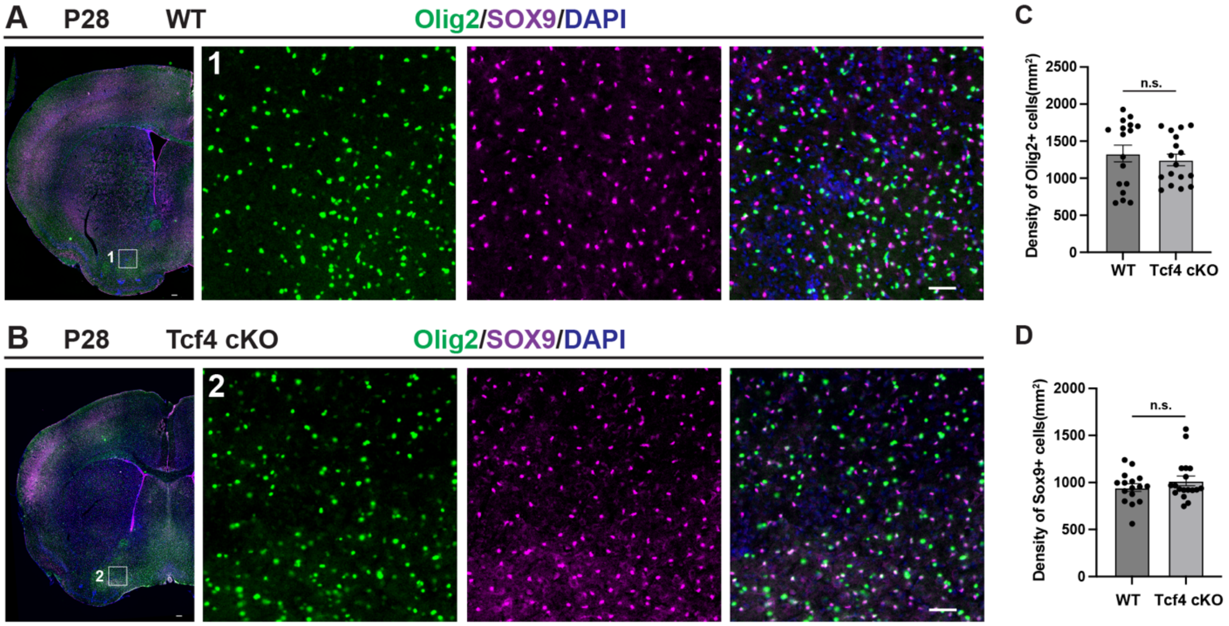

Appendix Figure S3. The density of Sox9-positive and Olig2-positive cells in the ventral brain of Tcf4 cKO mice are unchanged compared to those in WT mice. (A-B) Representative images of brain sections stained for Olig2 and Sox9 in WT brains (A) and Tcf4 cKO brains (B). The scale bar for the top left picture both in (A) and (B) is 200 μm, while the zoomed-in picutres on the right in (A) and (B) have a scale bar of 100 μm. (C-D) Quantification of the density of Olig2-positive cells (C) (n = 3 mice were analyzed for each genotype) and Sox9-positive cells (D) (n = 3 mice were analyzed for each genotype). Comparison between the two groups was conducted using a t-test within a linear mixed model. Error bars represent mean ± SEM. n.s., not significant.

Appendix Figure S4

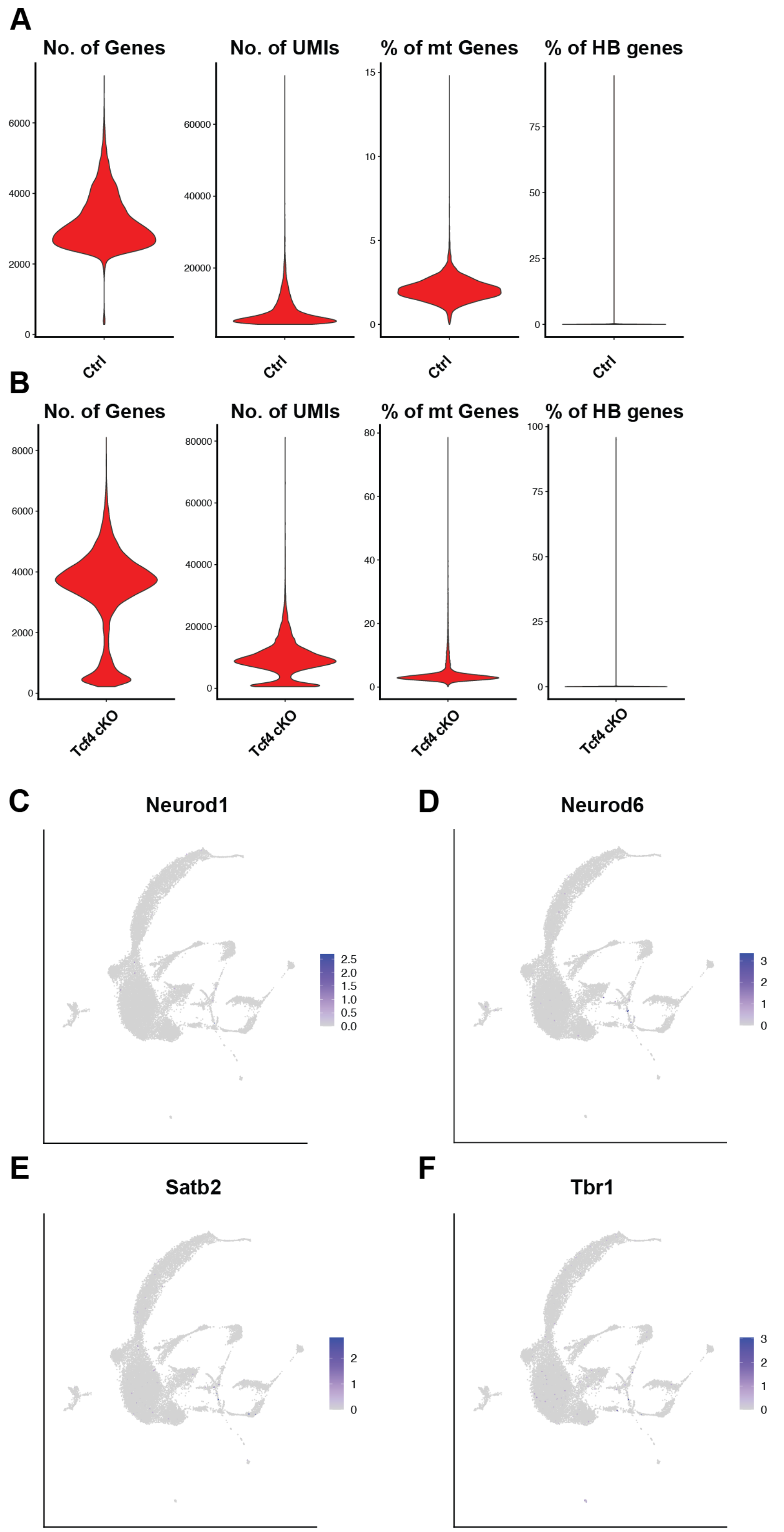

Appendix Figure S4. Quality control of single-cell RNA sequencing data from the Nkx2.1 lineage in the dorsal neocortex. (A-B) The number of genes, total unique molecules identified (UMIs) and the percentage of genes detected from mitochondrial and red blood cells in WT dataset (A) and Tcf4 cKO dataset (B). (C-F) Expression of projection neuronal markers such as Neurod1 (C), Neurod6 (D), Satb2 (E) and Tbr1 (F), shown in feature plot in the single-cell dataset in Fig.3 (B).

Appendix Figure S5

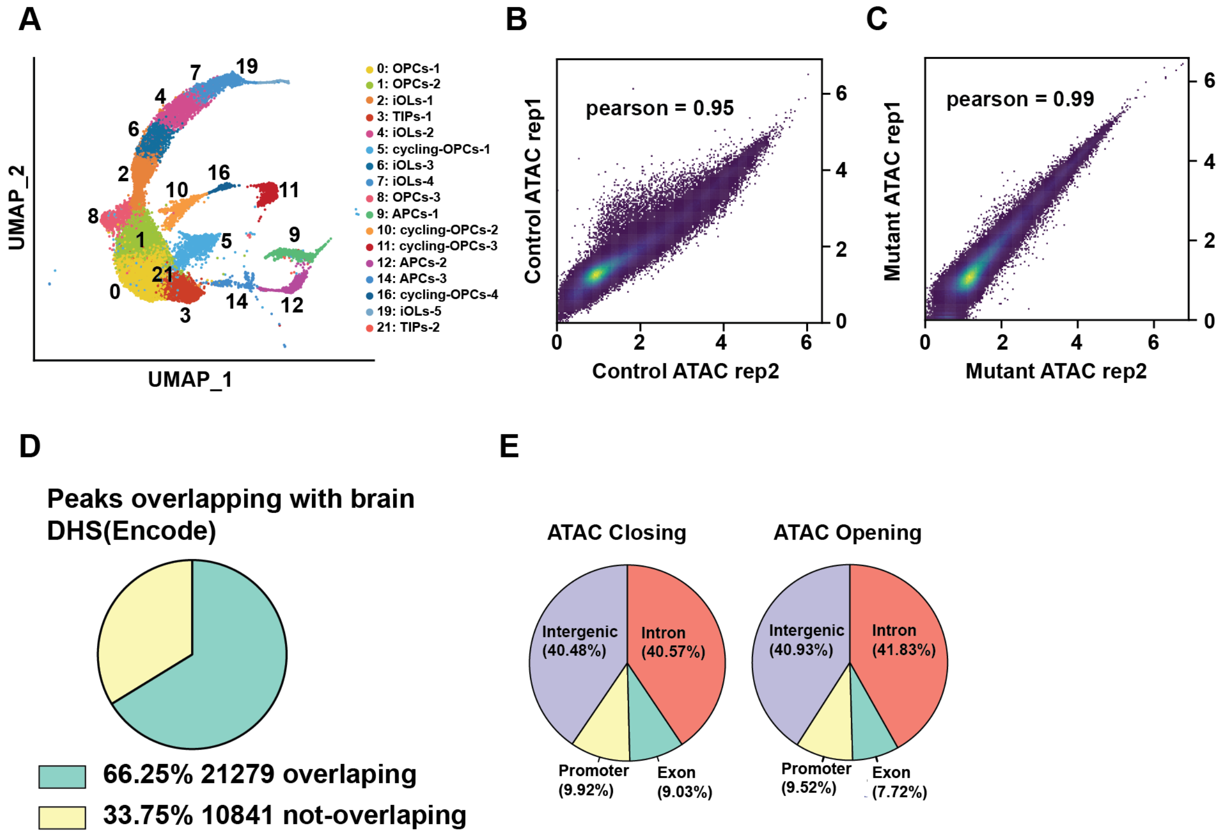

Appendix Figure S5. Depletion of Tcf4 in Nkx2.1-expressing radial glial cells enhances the accessibility of chromatin regions associated with genes related to astrogenesis. (A) UMAP visualization of subclusters in Figure 3. (B). Subclusters were used for lineage identification via Slingshot. (B-C) Pearson correlation analysis between experimental replicates of ATAC-seq data of WT (B) and Tcf4 cKO (C). (D) Quantification of the overlaps between ATAC-seq peaks and reference peaks from DNase-seq (an alternative method to assess chromatin accessibility) of the E18.5 mouse brain. (E) Quantification of the distribution of closing peaks and opening peaks relative to various genomic features.

Appendix Figure S6

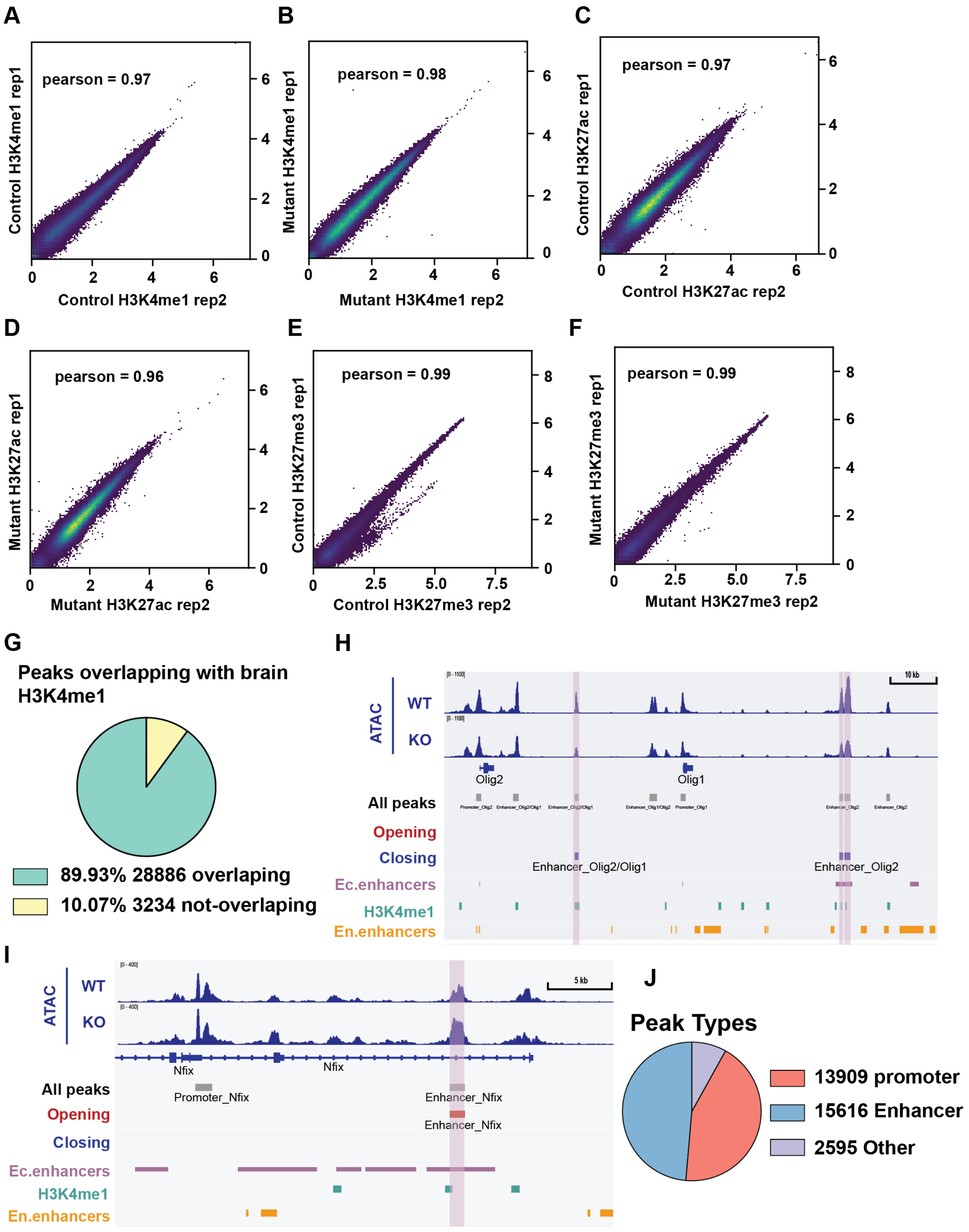

Appendix Figure S6. Deletion of Tcf4 in Nkx2.1-expressing RGCs alters epigenetic states of enhancers. (A-B) Pearson correlation between experimental replicates of Cut&Tag data for H3K4me1 in WT (A) and Tcf4 cKO (B). (C-D) Pearson correlation between experimental replicates of Cut&Tag data for H3K27ac in WT (C) and Tcf4 cKO (D). (E-F) Pearson correlation analysis between experimental replicates of Cut&Tag data for H3K27me3 in WT (E) and Tcf4 cKO (F). (G) Quantification of the overlaps between ATAC-seq peaks and H3K4me1peaks from the Cut&Tag assay of the Nkx2.1 lineage cells in the neocortex at P7. (H-I) Example track illustrating the peak assignment strategy in Figure 6A-6B. Enhancers are identified by overlapping with enhancers from Encode reference dataset (abbreviated as En.enhancers), the Ensemble regulatory build (including enhancers only, abbreviated as En.enhancers), and H3K4me1 peakJs. (J) Peaks assignments of total ATAC peaks.

Appendix Figure S7

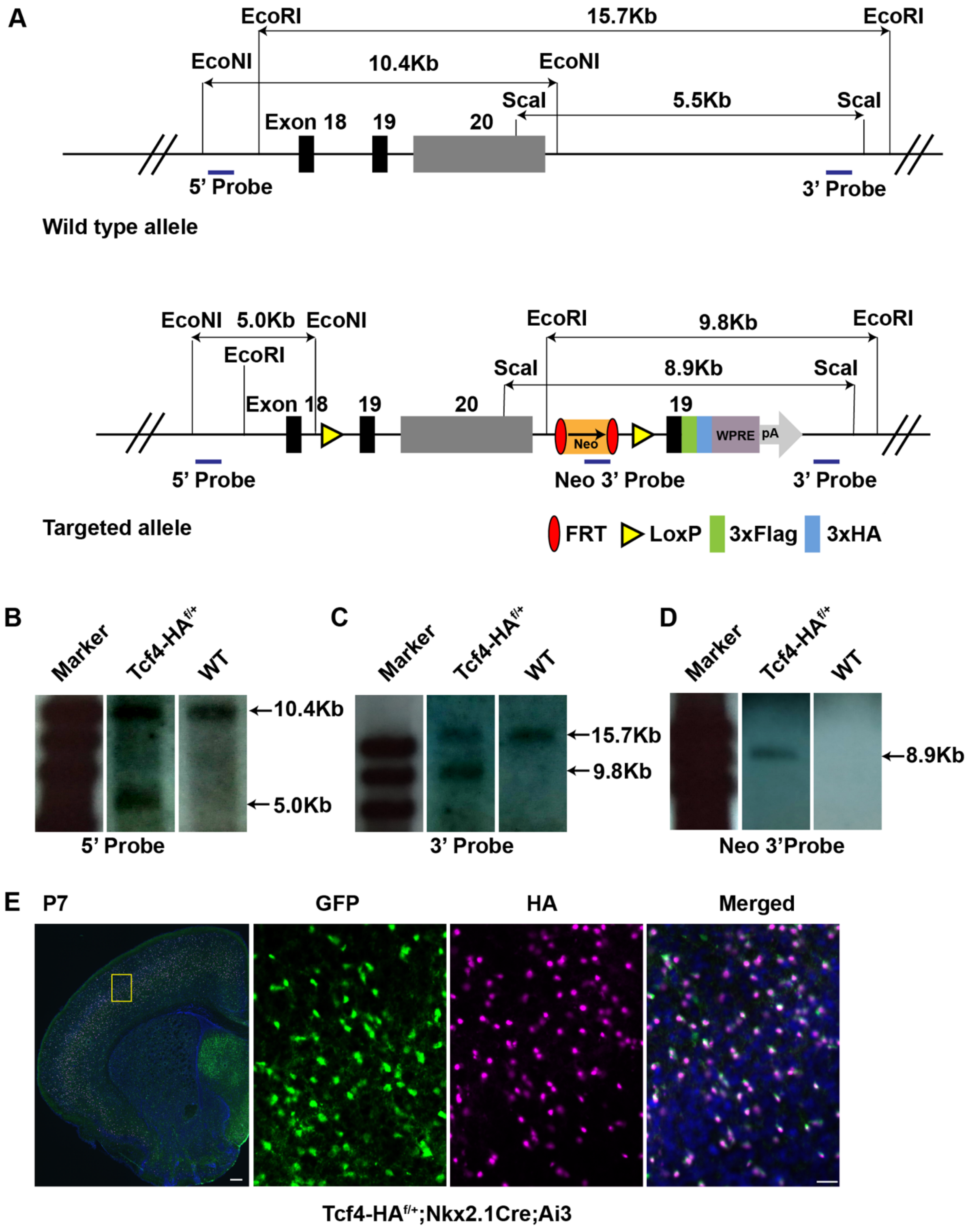

Appendix Figure S7. Design of Cre-dependent Tcf4-tag mice**.** (A) Scheme showing the design of Tcf4-tag mice. (B-D) Southern blot analysis probed with DNA fragments indicated 5'probe (B), 3'probe (C) and Neo 3'probe (D) to verify the correct insertion. (E) Representative images demonstrating HA-Tag is expressed in the Nkx2.1 lineage of the dorsal neocortex. Scale bar: 200μm in the left image and 100μm in the enlarged images.
