# Supplementary material for: Astrocyte allocation during brain development is controlled by Tcf4-mediated fate restriction
Source: EMBO J. 2024 Sep 19;43(21):5114–40. doi: 10.1038/s44318-024-00218-x (PMC11535398; doi:10.1038/s44318-024-00218-x)
Supplement: Supplementary file 3 — Expanded View Figures [file 44318_2024_218_MOESM3_ESM.pdf]

Expanded View Figures

Figure EV1. Astrocyte allocation in dorsal neocortex and ventral telencephalon.

(**A**) Sparse labeling of Emx1 lineage cells induced at E12.5 reveals no astrocytes from the Emx1 lineage observed in the ventral telencephalon at P21. However, in the dorsal neocortex, astrocytes can be traced from the Emx1 lineage generated at E12.5. Sox9 and GFP double-positive astrocytes are indicated by yellow arrowheads. Scale bar: 50 µm. (**B**) Sparse labeling of Nkx2.1 lineage cells induced at E12.5 shows no astrocytes from Nkx2.1 lineage observed in the dorsal neocortex at P21. However, in the ventral telencephalon, astrocytes can be traced from the Nkx2.1 lineage generated at E12.5. Yellow arrowheads indicate Sox9 and GFP double-positive astrocytes. Scale bar: 50 µm. (**C**) A schematic diagram illustrates the location of astrocytes derived from the Emx1 and Nkx2.1 lineages.

© The Author(s)

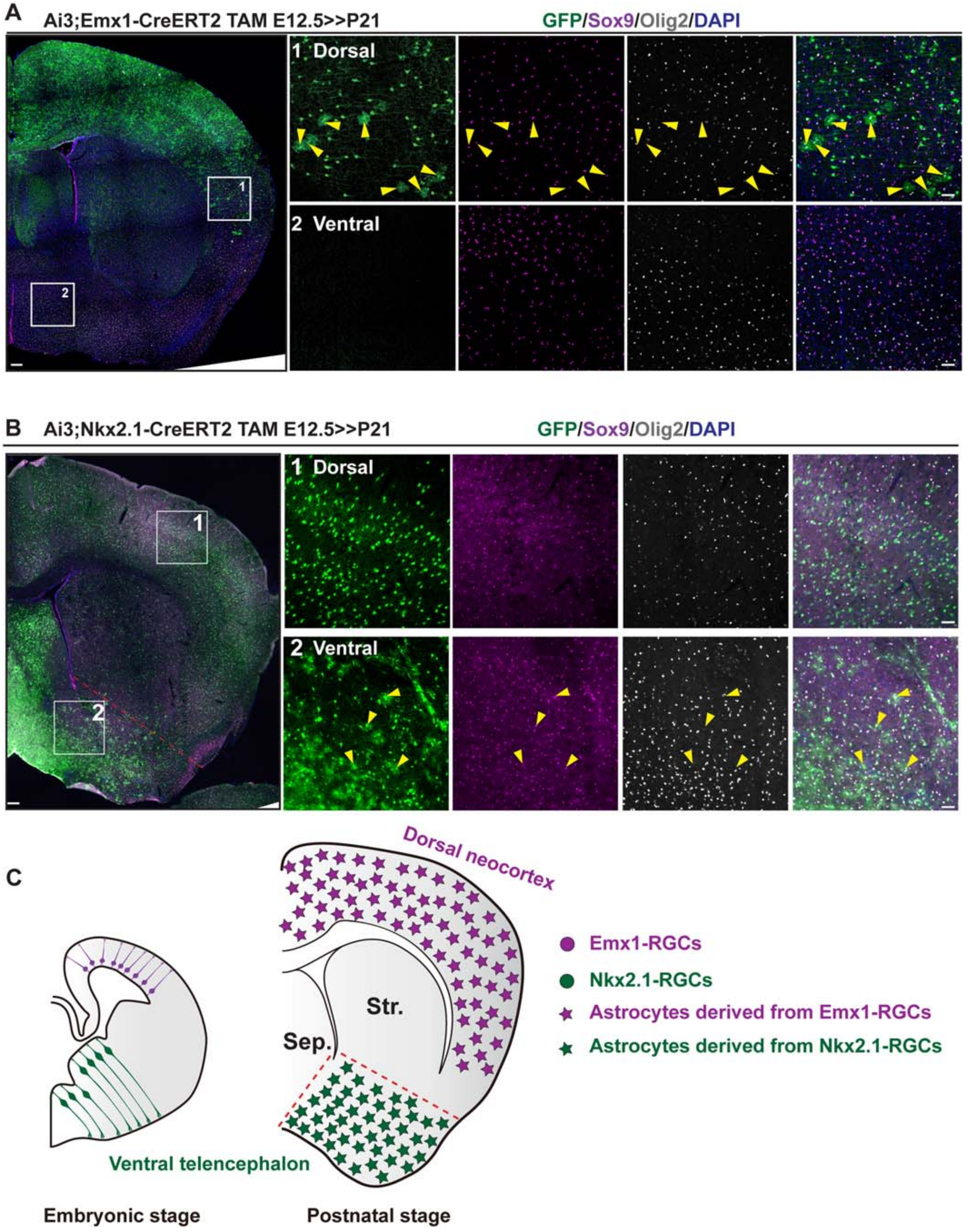
A
Ai3;Emx1-CreERT2 TAM E12.5>>P21
GFP/Sox9/Olig2/DAPI
1 Dorsal
2 Ventral
B
Ai3;Nkx2.1-CreERT2 TAM E12.5>>P21
GFP/Sox9/Olig2/DAPI
1 Dorsal
2 Ventral
C
Dorsal neocortex
Str.
Sep.
Ventral telencephalon
Embryonic stage
Postnatal stage
Emx1-RGCs
Nkx2.1-RGCs
Astrocytes derived from Emx1-RGCs
Astrocytes derived from Nkx2.1-RGCs

 © The Author(s)

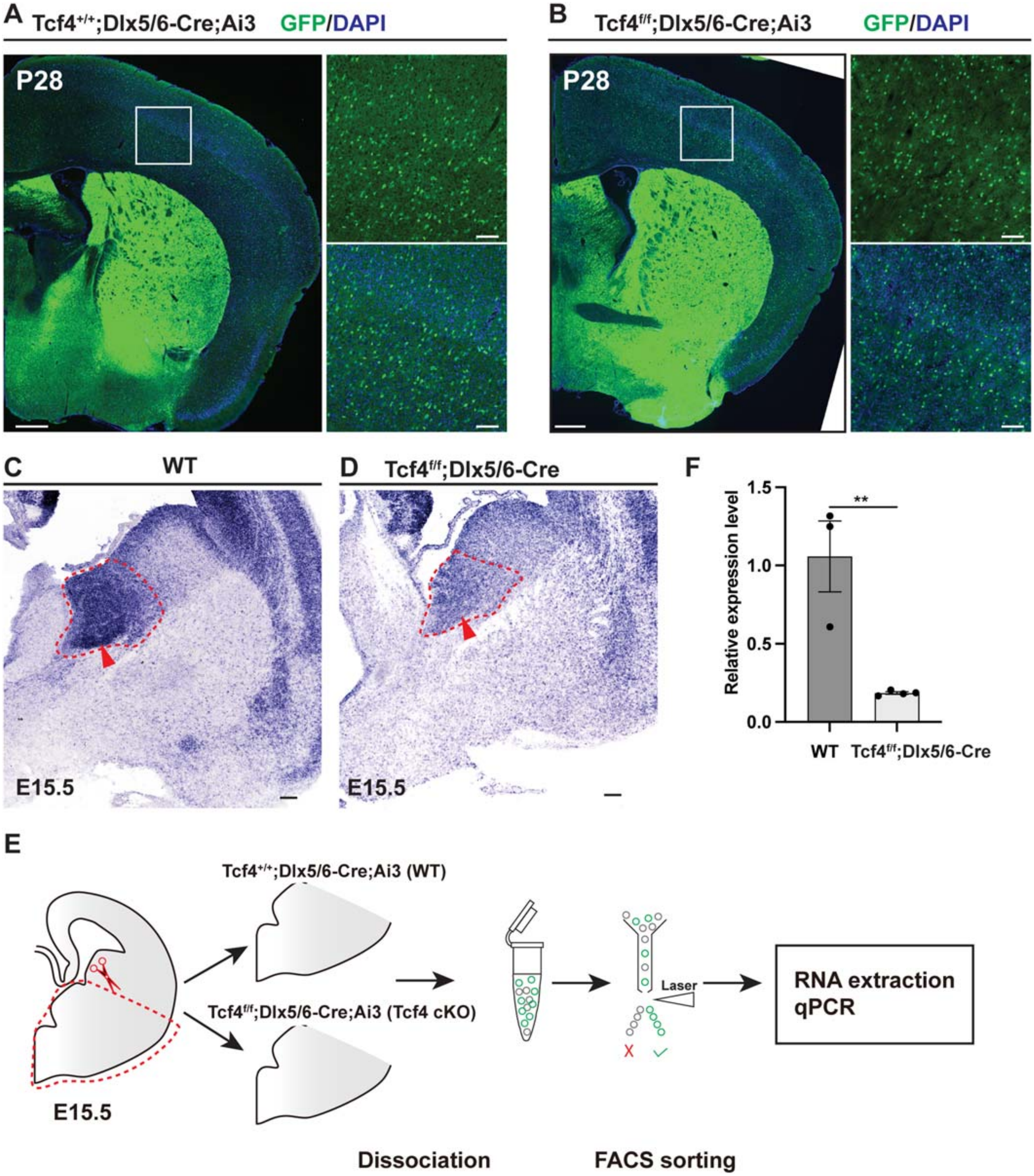
A Tcf4+/+;Dlx5/6-Cre;Ai3 GFP/DAPI
P28
B Tcf4f/f;Dlx5/6-Cre;Ai3 GFP/DAPI
P28
C WT
E15.5
D Tcf4f/f;Dlx5/6-Cre
E15.5
F
Relative expression level
1.5
1.0
0.5
0.0
**
WT
Tcf4f/f;Dlx5/6-Cre
E
Tcf4+/+;Dlx5/6-Cre;Ai3 (WT)
Tcf4f/f;Dlx5/6-Cre;Ai3 (Tcf4 cKO)
E15.5
Laser
RNA extraction
qPCR
Dissociation
FACS sorting

◀ **Figure EV2. Ectopic astrocytes in the dorsal neocortex do not arise from committed progenitors.**

(**A**) Representative images of brain sections from Tcf4$^{+/+}$; Dlx5/6-Cre mice stained for GFP at P28. (**B**) Representative images of brain sections from Tcf4$^{f/f}$; Dlx5/6-Cre mice stained for GFP at P28. Scale bars represent 500 μm and 100 μm respectively. (**C**, **D**) Expression of *Tcf4* was examined by in situ hybridization in the brains of WT and Tcf4$^{f/f}$; Dlx5/6-Cre mice at E15.5. The MGE region is circled with red lines, the red arrowheads indicate the region within which the expression of Tcf4 is decreased, scale bar: 100 μm. (**E**) A schematic diagram illustrates the procedure of collecting the GFP+ cells from WT and Tcf4$^{f/f}$; Dlx5/6-Cre mice and performing real-time quantitative PCR experiments (qPCR). (**F**) The relative expression level of *Tcf4* ($n = 3$ embryos for WT, and $n = 4$ embryos for Tcf4$^{f/f}$; Dlx5/6-Cre). Student's T-test using linear mixed model is used for comparing two groups. $p = 0.0058$. Error bars represent mean ± SEM. $^{**}p < 0.01$.

 © The Author(s)

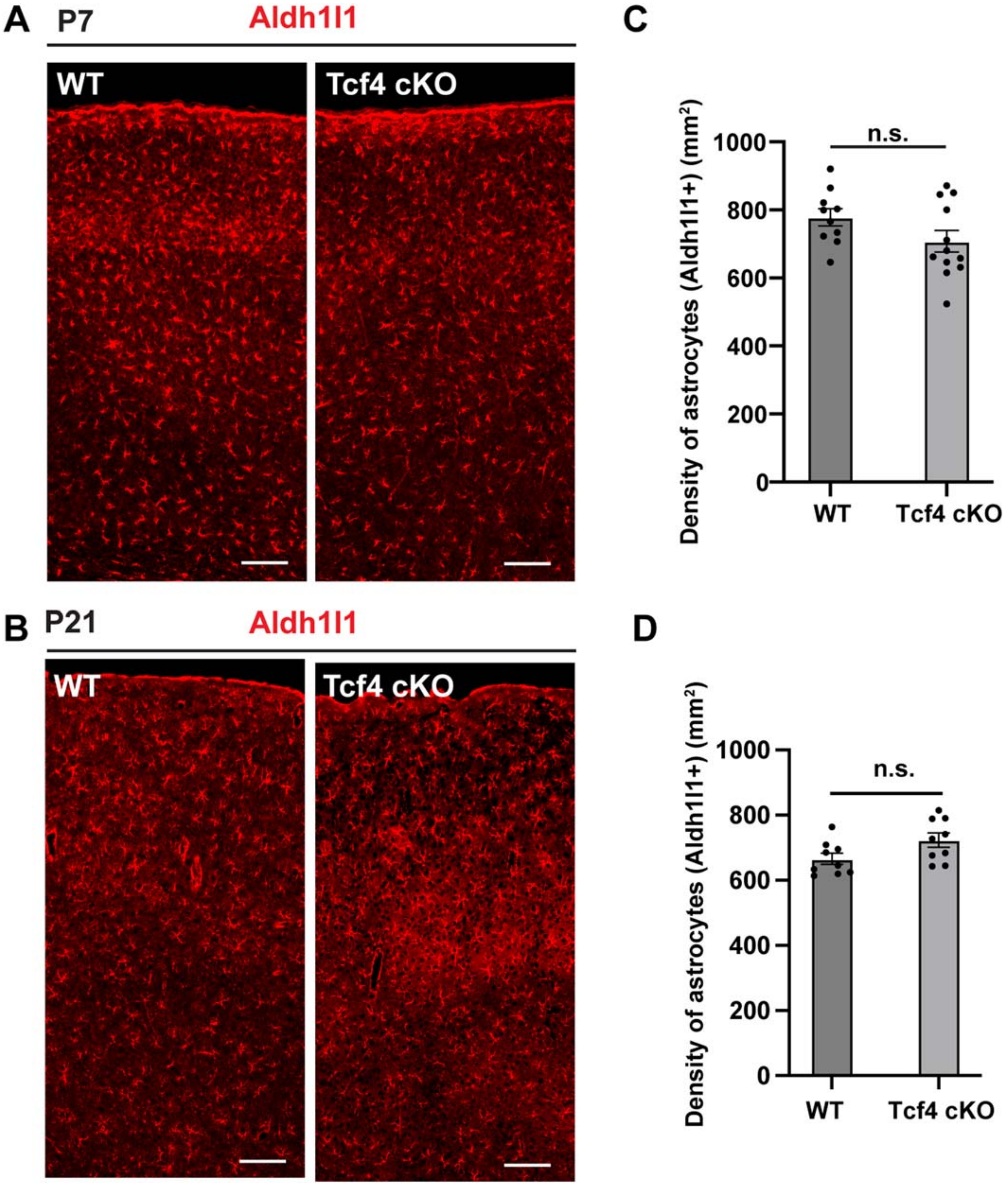
A
P7
Aldh1l1
WT
Tcf4 cKO
C
Density of astrocytes (Aldh1l1+) (mm²)
1000
800
600
400
200
0
n.s.
WT
Tcf4 cKO
B
P21
Aldh1l1
WT
Tcf4 cKO
D
Density of astrocytes (Aldh1l1+) (mm²)
1000
800
600
400
200
0
n.s.
WT
Tcf4 cKO

© The Author(s)

Figure EV3. The density of Aldh1l1-positive astrocytes in the neocortex was unchanged between Tcf4 cKO and WT brains.

(**A**, **B**) Representative images of brain sections stained for Aldh1l1 in WT and Tcf4 cKO at P7 (**A**) and at P21 (**B**), Scale bar: 100 µm. (**C**, **D**) Quantification of the density of Aldh1l1+ astrocytes in the neocortex at P7 (**C**), and at P21 (**D**) ($n = 3$ mice were analyzed for each genotype). Comparison between the two groups was conducted using a t-test within a linear mixed model. Error bars represent mean ± SEM.

 © The Author(s)

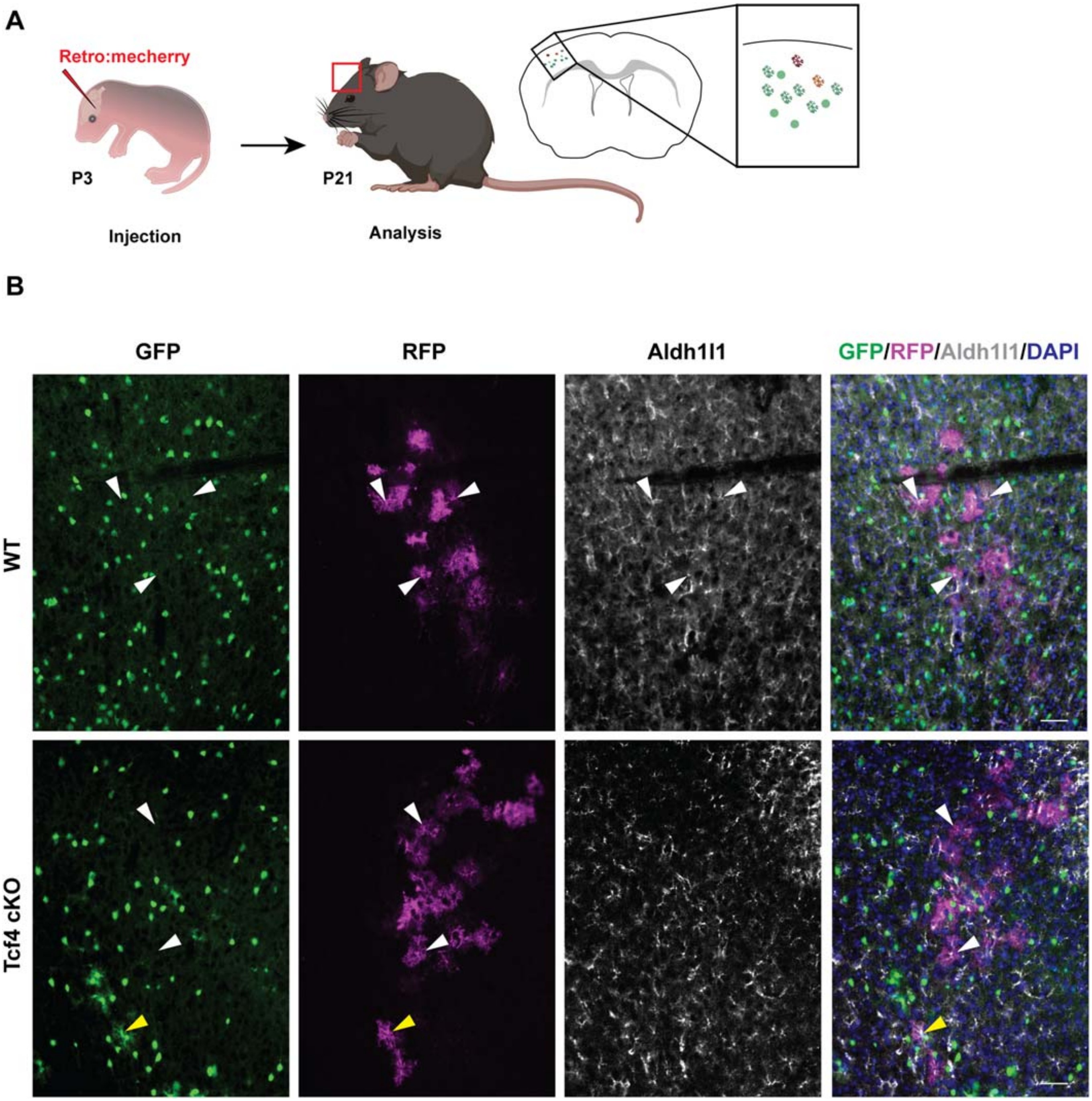

Figure EV4. Astrocyte precursor cells generate ectopic astrocytes in the dorsal neocortex of Tcf4 cKO brain.

(**A**) A schematic diagram illustrates the experimental design. (**B**) Representative images of brain sections in WT and Tcf4 cKO brains stained for GFP, RFP, and Aldh1l1. In WT brains, no RFP-labeled astrocytes co-labeled with GFP were observed, while GFP+ astrocytes in the dorsal neocortex of Tcf4 cKO brains were labeled with RFP. Yellow and white arrowheads indicate astrocytes labeled by retrovirus that are GFP-positive or GFP-negative respectively. Scale bar, 100 µm.

© The Author(s)
